# A Grand Opportunity

**Published:** 1887-09

**Authors:** 


A GRAND OPPORTUNITY.—Homœopathy claims to be *the* science of therapeutics. So wrote Carroll Dunham, and in proof of this claim he clearly demonstrated that Homœopathy, and Homœopathy only of all medical theories, fulfilled the two great conditions of a science. These two conditions or tests of a science Dunham declared were—(1) "A capability of infinite progress in each of its elements without detriment to its integrity;" (2) that "it must furnish means of *prevision.*" Homœopathy only fulfilled these tests, said Dr. Dunham. Another witness, Dr. Constantine Hering, was certainly the peer of any physician of the century—a man of the greatest ability combined with fullest medical knowledge, ripened by the practical experience of a half century, and Dr. Hering believed Homœopathy was *the* science of therapeutics. Samuel Hahnemann declared Homœopathy to be the *one* method of cure; Hering and Dunham have borne witness to the truth of Hahnemann's declaration. We do not call upon the thousands of successful practitioners of this law to prove its value; their testimony would be unanimous in its favor.

We merely assert that any statement made by such a man as Hahnemann, and corroborated by such men as Hering and Dunham, is entitled to the greatest respect; that such testimony can only be disproven by the weightiest evidence, and that this evidence should be given by able men and corroborated by ample proof. The allopaths have for years sillily ignored or abusively calumniated the evidence for a great therapeutic truth given by able and honest men. This was to be expected. But that those of the same medical school as were Hering and Dunham should so slight their testimony was *not* to be expected. For a long time these doubters in the homœopathic school have declared that Homœopathy was not able to cope with all diseases; that it was merely *one* of many methods of treating disease. It has been said there "*are* logical and scientific reasons" for deserting homœopathic practice for "other measures." These declarations have been made from time to time by irresponsible nobodies who could not prove their asseverations.

But lately the New York *Med. Times* quotes Dr. T. F. Allen as saying: "We do not hold Homœopathy is the exclusive and the only law of healing. It is the most widely and generally useful, but not the only guide in the treatment of the sick." This is a definite, clear statement, made by a physician who is a prominent teacher, an author, and a man of great ability. Dr. Allen is not the man to make idle or ill-considered assertions. If he has made this declaration he is ready and doubtless able to give proof for the "faith that is in him." We can only hope that he will give the profession his reasons and his proof for this statement. As stated before, Hahnemann believed Homœopathy to be the *one* curative method; he believed it to be able to cure all curable diseases, and in incurable diseases, even, to afford more comfort and better aid than any of the so-called palliatives. Now what we ask Dr. Allen to tell us, is, where Homœopathy fails, and what is the other "guide in the treatment of the sick"? If Homœopathy be only "the most widely and generally useful" guide, let us have the other.

Then our knowledge of medicine will be complete. We will have guides for all possible cases, and the man who can so complete the system of medicine will be a second Hahnemann, whom all will delight to honor. It is a grand opportunity.

